# Data-Driven Design of PROTAC Linkers to Improve PROTAC Cell Membrane Permeability

**DOI:** 10.1021/jacsau.6c00033

**Published:** 2026-02-09

**Authors:** Yuki Murakami, Shoichi Ishida, Nobuo Cho, Hitomi Yuki, Masateru Ohta, Teruki Honma, Yosuke Demizu, Kei Terayama

**Affiliations:** † Graduate School of Medical Life Science, 13155 Yokohama City University, 1-7-29, Suehiro-cho, Tsurumi-ku, Yokohama, Kanagawa 230-0045, Japan; ‡ RIKEN Center for Integrative Medical Sciences, 1-7-22, Suehiro-cho, Tsurumi-ku, Yokohama, Kanagawa 230-0045, Japan; § HPC- and AI-driven Drug Development Platform Division, RIKEN Center for Computational Science, 1-7-22, Suehiro-cho, Tsurumi-ku, Yokohama, Kanagawa 230-0045, Japan; ∥ Division of Organic Chemistry, National Institute of Health Sciences, 3-25-26, Tonomachi, Kawasaki-ku, Kawasaki, Kanagawa 210-9501, Japan; ⊥ RIKEN Center for Advanced Intelligence Project, 1-4-1, Nihonbashi, Chuo-ku, Tokyo 103-0027, Japan; # School of Life Science and Technology, Institute of Science Tokyo, 4259, Nagatsuta-cho, Midori-ku, Yokohama, Kanagawa 226-8501, Japan

**Keywords:** PROTAC, cell membrane permeability, linker design, machine learning, molecular generation

## Abstract

Proteolysis-targeting chimeras (PROTACs) are promising next-generation therapeutics for the degradation of disease-associated proteins. However, optimizing the physicochemical properties of PROTACs, particularly their poor cell membrane permeability, remains challenging. Traditionally, PROTAC linkers have been manually designed to improve cell membrane permeability. Although recent machine learning-based approaches have enabled the rational design of PROTAC linkers, no linker design methods that explicitly address cell membrane permeability have been reported. In this study, we developed PROTAC-TS, a linker generative model that combines a chemical language model and reinforcement learning to control cell membrane permeability. We first constructed a prediction model of cell membrane permeability, which achieved high prediction performance (*R* ^2^ = 0.710). By integrating this prediction model into the generative model, we successfully designed linkers of PROTACs with high predicted cell membrane permeability while considering PROTAC likeness. Our results highlight the potential of PROTAC-TS in accelerating PROTAC development with favorable cell membrane permeability.

## Graphical abstract

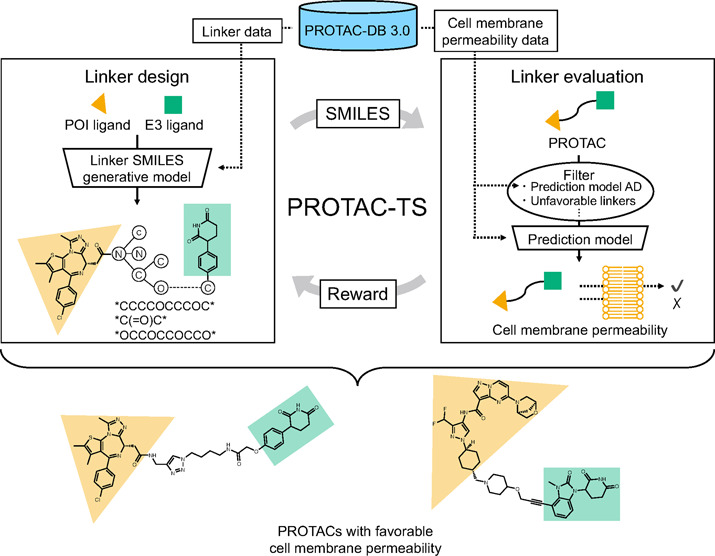

## 1 Introduction

Proteolysis-targeting chimeras (PROTACs) have garnered significant interest as next-generation therapeutics capable of degrading disease-associated proteins of interest (POIs).^1–3^ PROTACs are heterobifunctional molecules comprising an E3 ligase-binding moiety (E3 ligand), POI-binding ligand, and linker. This unique architecture enables PROTACs to form degradation-competent ternary complexes with E3 ligases and POIs, thereby catalytically inducing POI degradation by hijacking the ubiquitin–proteasome system (UPS).^4^ Furthermore, they have the potential to target disease-associated proteins that lack conventional ligand-binding pockets and have been considered undruggable because PROTACs rely on the induced proximity between a POI and an E3 ligase rather than binding to a functional active site. Currently, several PROTACs, such as ARV-110 ^5^ and ARV-471,^6^ are being evaluated in clinical trials.^7^

Despite their promise, optimizing the physicochemical properties of PROTACs remains a significant challenge, especially their poor cell membrane permeability,^8^ which leads to poor bioavailability and an increased risk of preclinical or clinical failure.^9^ Cell membrane permeability is one of the factors determining oral bioavailability alongside solubility and first-pass metabolism.^10^ Most PROTACs fall beyond the rule of five (bRo5) chemical space because of their larger molecular size compared to that of conventional inhibitors and, consequently, exhibit low cell membrane permeability.^11–13^ As PROTACs induce protein degradation via intracellular UPS, low cell membrane permeability reduces degradation activity.^14^ Therefore, improving the cell membrane permeability of PROTACs is essential for achieving sufficient pharmacological effects. High cell membrane permeability also allows lower doses to achieve effective intracellular concentrations and contributes to a reduced risk of toxicity.

To date, the cell membrane permeability of PROTACs has typically been controlled by optimizing their linkers based on a traditional manual design.^15^ Linkers are instrumental in determining the key properties of PROTACs, including cell membrane permeability and protein degradation activity.^16–24^ For instance, Klein et al. improved both the cell membrane permeability and degradation activity of MZ1 and ARV-771, which are bromodomain and extra-terminal PROTACs, by replacing the amide bonds with esters to reduce the number of hydrogen bond donors and increase hydrophobicity.^16^ Abeje et al. designed nine von Hippel-Lindau (VHL)-based PROTACs with different linker structures and evaluated their cell membrane permeability.^25^ They found that PROTACs with stable conformations formed through intramolecular interactions, including NH−π and π–π interactions under low-polarity conditions, exhibited higher cell membrane permeability. However, empirical rule-based manual design still requires substantial time and effort for linker optimization, leading to a bottleneck in the PROTAC development cycle.^26^ Improving the efficiency of the linker optimization process is expected to shorten the development timeline of PROTACs.

Recently, machine learning (ML)-based linker design methods that account for various molecular properties have been developed.^27–34^ For instance, Tan et al. developed DRlinker, which incorporates the linker length and log*P*.^29^ Neeser et al. proposed ShapeLinker, which considers the linker length, number of rotatable bonds, and linker conformation within a ternary complex.^28^ Li et al. introduced PROTAC-INVENT, which integrates both two-dimensional (2D) features, such as the number of aromatic rings in a linker, and three-dimensional (3D) features, such as the docking scores of ternary complexes.^30^ Zheng et al. developed PROTAC-RL, which employs the AbbVie multiparametric score, a metric indicative of oral absorption in the bRo5 space, as a design guideline.^31^ Despite these advances, PROTAC linker design methods that explicitly consider cell membrane permeability have yet to be reported.

One possible reason for the absence of such methods is the lack of open-source tools for assessing the cell membrane permeability of PROTACs. Although several previous studies developed ML-based models to predict this property, some limitations remain. For instance, although Poongavanam et al. developed a classification model for the cell membrane permeability of PROTACs,^35^ it was trained on an in-house data set and not publicly available. Peteani et al. developed regression models for absorption, distribution, metabolism, excretion, and physicochemical properties, including the cell membrane permeability of PROTACs (low-efflux Madin–Darby canine kidney cell line *P*_app_, *R* = 0.971), and provided access to the source code; however, the experimental data used for model training were not disclosed, making it impossible to reproduce the prediction performance reported in their study.^36^ Thus, the lack of accessible and high-performance tools for evaluating the cell membrane permeability of PROTACs may constitute a key bottleneck in the development of linker design methods that account for this property. Although public databases for PROTACs, such as PROTAC-DB 3.0,^37^ have been established, their small data set sizes make it difficult to handle extrapolated data when used for ML-based model development.

In this study, we developed a PROTAC linker design method called PROTAC-TS, which considers cell membrane permeability. To evaluate the cell membrane permeability of PROTACs, we first constructed a prediction model based on PROTAC-DB 3.0. The prediction model achieved high prediction performance (coefficient of determination (*R* ^2^) = 0.710, Pearson correlation coefficient (*R*) = 0.846, and root-mean-square error (RMSE) = 0.517) and is publicly available. Subsequently, we developed PROTAC-TS by implementing a linker generation function in ChemTSv2,^38^ a de novo molecule generator based on reinforcement learning, and integrated the prediction model to modulate cell membrane permeability. PROTAC-TS can be used to design PROTAC linkers while considering PROTAC likeness, including structural stability, synthetic accessibility, structural branching, and linker length, by applying filters. Furthermore, to address the problem of extrapolated data, PROTAC-TS considers the applicability domain (AD) of the prediction model during linker design, thereby ensuring reliable predictive performance by focusing on data close to the training data.^39–41^ PROTAC-TS successfully designed linkers for PROTACs with high predicted cell membrane permeability for several POI–E3 ligand pairs. It also reproduced a linker for a PROTAC with high cell membrane permeability, as listed in PROTAC-DB 3.0, and the linker of KT-474, a PROTAC that has reached phase 2 clinical trials.^42^ Additionally, experimental evaluations confirmed the high cell membrane permeability of PROTACs containing linkers designed by PROTAC-TS. These results indicate the potential of PROTAC-TS to accelerate the development of PROTACs with improved cell membrane permeability. The source code is available at https://github.com/ycu-iil/PROTAC-TS.

## 2 Results and Discussion

Initially, we constructed prediction models for cell membrane permeability of PROTACs by using a tabular prior-data fitted network (TabPFN).^43^ Subsequently, we developed PROTAC-TS by integrating ChemTSv2 with the best-performing prediction model (Figure 1) and applied the method to design PROTAC linkers. The adaptability and effectiveness of PROTAC-TS were further confirmed by reproducing the linker of a PROTAC with high cell membrane permeability listed in PROTAC-DB 3.0 and the linkers of PROTACs that have entered clinical trials. Finally, we experimentally validated the cell membrane permeability of several designed PROTACs.

**Figure 1:**
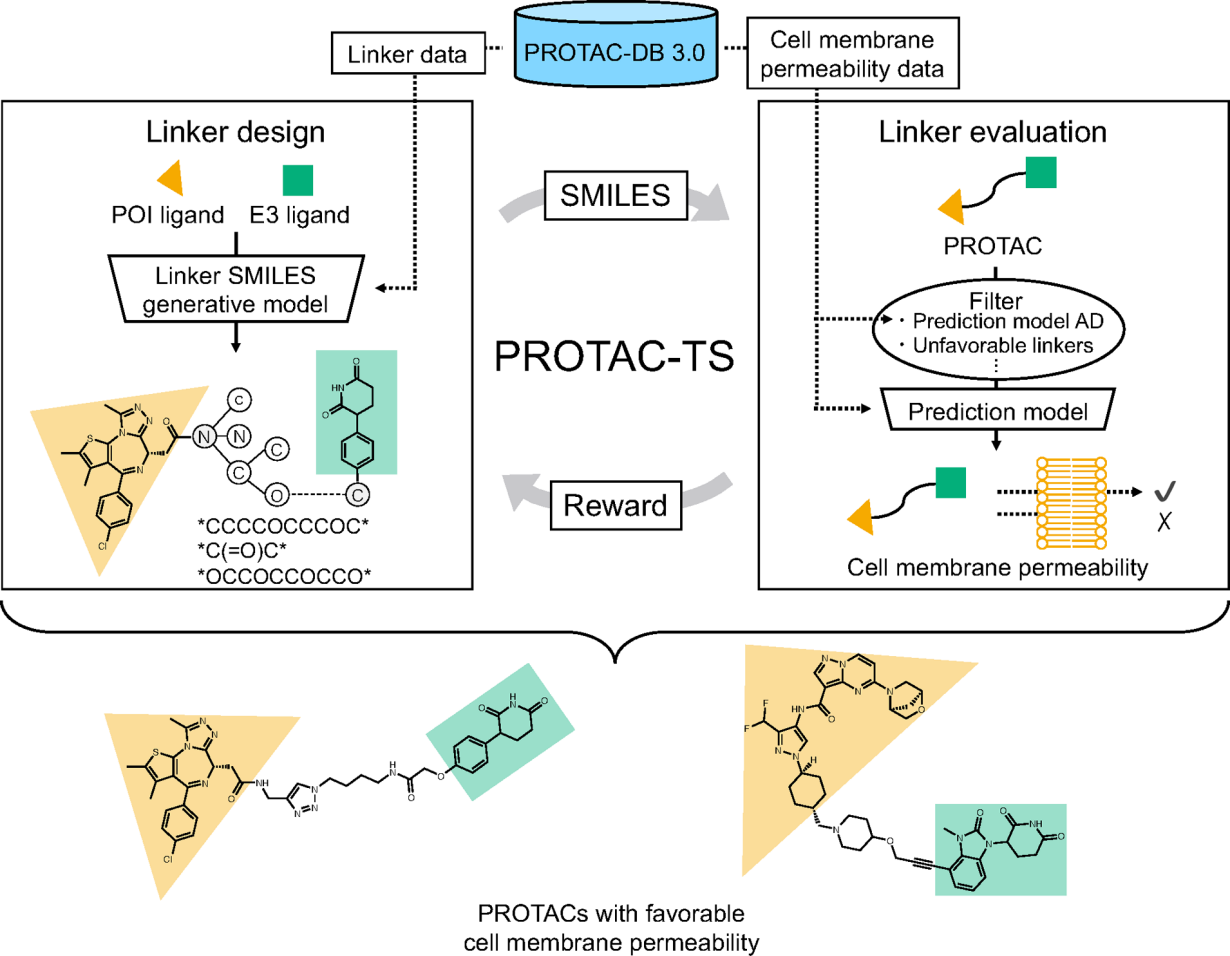
Overview of the PROTAC-TS workflow. The workflow comprises two main modules: “Linker design” and “Linker evaluation”. In the “Linker design” module, a generative model, which was trained on the PROTAC-DB 3.0 linker data set, generates SMILES for linkers that connect a selected POI and E3 ligand. In the “Linker evaluation” module, the generated linkers are first passed through predefined filters. The cell membrane permeability of the filtered linkers is then predicted using a prediction model trained on the PROTAC-DB 3.0 data set. A reward is calculated based on the predicted cell membrane permeability and fed back to the “Linker design” module. By repeating this cycle, PROTAC-TS designs linkers of PROTACs with favorable cell membrane permeability.

### 2.1 Construction of Prediction Models for Cell Membrane Permeability

We constructed prediction models for the cell membrane permeability of PROTACs using PROTAC-DB 3.0 (described in Section 4.1). Various features and prediction methods were evaluated (Table 1). The features included Mordred descriptors containing only 2D descriptors, Mordred descriptors containing 3D descriptors, bit-based Morgan fingerprints, 2048-dimensional count-based Morgan fingerprints, and 500-dimensional count-based Morgan fingerprints. The prediction methods included TabPFN, light gradient boosting machine (LightGBM),^44^ and AutoGluon.^45^ Among all the combinations, the prediction model employing the 500-dimensional count-based Morgan fingerprint as the feature and TabPFN as the prediction method achieved the highest prediction performance, as shown in Figure 2 (*R* ^2^ = 0.710, *R* = 0.846, and RMSE = 0.517). Across all methods, the prediction models using Mordred descriptors containing 3D descriptors consistently outperformed those using Mordred descriptors containing only 2D descriptors. For the prediction models based on Morgan fingerprints, both TabPFN and AutoGluon exhibited higher performance using count-based Morgan fingerprints than bit-based Morgan fingerprints. This result likely reflects the ability of count-based Morgan fingerprints to capture repetitive structural patterns frequently found in linkers containing polyethylene glycol or alkane moieties. Based on thesed results, we constructed a prediction model using a combination of features and methods that achieved the best performance. The model, trained on 43 compounds from PROTAC-DB 3.0, was employed to evaluate the cell membrane permeability of PROTACs to guide linker design.

**Table 1:** Performance of the Prediction Models for Caco-2 Cell Membrane Permeability^a^

model	feature	dimension	*R* ^2^	*R*	RMSE
TabPFN	Mordred (2D)	1422	0.617	0.788	0.594
TabPFN	Mordred (2D and 3D)	1473	0.636	0.802	0.579
TabPFN	Morgan (bit-based)	2048	0.646	0.808	0.571
TabPFN	Morgan (count-based)	2048	0.661	0.816	0.559
TabPFN	Morgan (count-based)	500	**0.710**	**0.846**	**0.517**
LightGBM	Mordred (2D)	1422	0.388	0.662	0.751
LightGBM	Mordred (2D and 3D)	1473	0.426	0.679	0.727
LightGBM	Morgan (bit-based)	2048	0.605	0.787	0.603
LightGBM	Morgan (count-based)	2048	0.599	0.776	0.608
LightGBM	Morgan (count-based)	500	0.649	0.814	0.568
AutoGluon	Mordred (2D)	1422	0.459	0.697	0.706
AutoGluon	Mordred (2D and 3D)	1473	0.557	0.749	0.639
AutoGluon	Morgan (bit-based)	2048	0.339	0.626	0.780
AutoGluon	Morgan (count-based)	2048	0.484	0.716	0.689
AutoGluon	Morgan (count-based)	500	0.548	0.750	0.645

a The models were developed to predict the log_10_-transformed experimental A-to-B permeability using PROTAC SMILES as input. Values shown in bold indicate the best-performing results.

**Figure 2:**
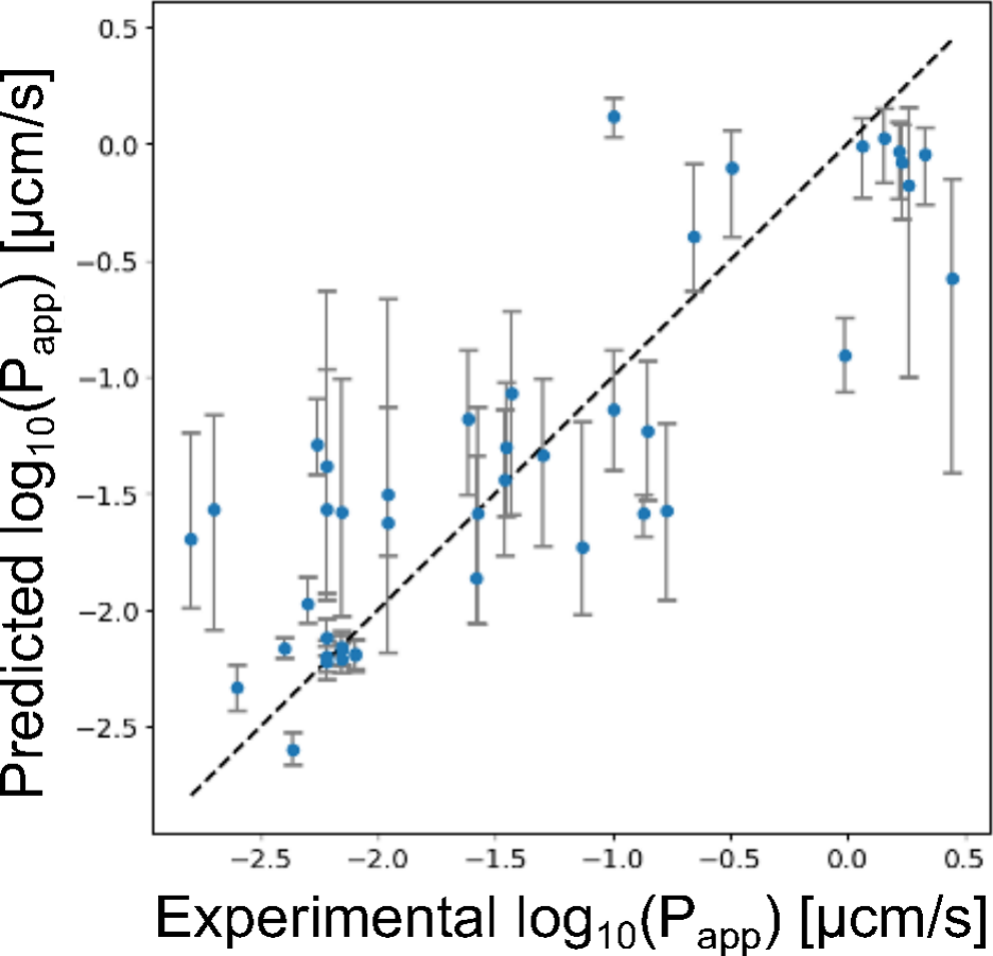
Scatterplot showing predicted vs actual cell membrane permeability. The points represent the mean of the predictive distribution from the best-performing prediction model (*R* ^2^ = 0.710, *R* = 0.846, and RMSE = 0.517). The upper and lower error bars correspond to the 70th and 30th percentiles of the predictive distribution, respectively.

### 2.2 Computational Validation for PROTAC-TS

#### 2.2.1 PROTAC Linker Design Based on Bromodomain-Containing Protein 4 (BRD4) and VHL Ligands

PROTAC linkers were designed to improve the cell membrane permeability of PROTACs based on BRD4 and VHL ligands.^46,47^ PROTAC-TS designs linkers using a recurrent neural network (RNN)-based linker generator in combination with chemical space exploration via Monte Carlo Tree Search (MCTS), as described in Section 4.2. The RNN-based linker generator was trained on the PROTAC linkers collected from PROTAC-DB 3.0. The BRD4 and VHL ligands, both commonly used in PROTAC design, were selected as the POI and E3 ligands for linker design, respectively, as shown in Figure 3a. PROTAC-TS incorporates various filters during linker design to consider PROTAC likeness. In this study, we examined three filtering conditions: relaxed, intermediate, and strict. The relaxed condition was applied only to the minimal filters. The intermediate condition included additional filters that excluded structurally unfavorable linkers, including those that were unstable, synthetically challenging, or excessively branched. The strict condition included further filters based on the AD of the prediction model, the structural similarity to the PROTAC linkers in PROTAC-DB 3.0, and linker length. Under the relaxed condition, the reward values improved progressively during the design process, as shown in Figure 3b. However, many of the designed linkers contained unstable or synthetically challenging substructures, as shown in Figure S1. Under the intermediate condition, PROTAC-TS designed PROTAC linkers with fewer unstable, synthetically challenging, or excessively branched substructures, as shown in Figure S2. Under both the relaxed and intermediate conditions, structural complexity metrics, such as linker length, the number of heteroatoms, the number of rotatable bonds, and the number of ring structures, increased with the linker generation, as shown in Figure S3. Under the strict condition, the designed linkers exhibited a lower incidence of undesired structures and greater similarity to the training data, as shown in Figures 3c and S4. This condition suppressed the increase in structural complexity metrics during the generation process, as shown in Figure S3. These results demonstrate that PROTAC-TS enables the control of the chemical space of the designed linkers by appropriately adjusting the filtering conditions.

**Figure 3:**
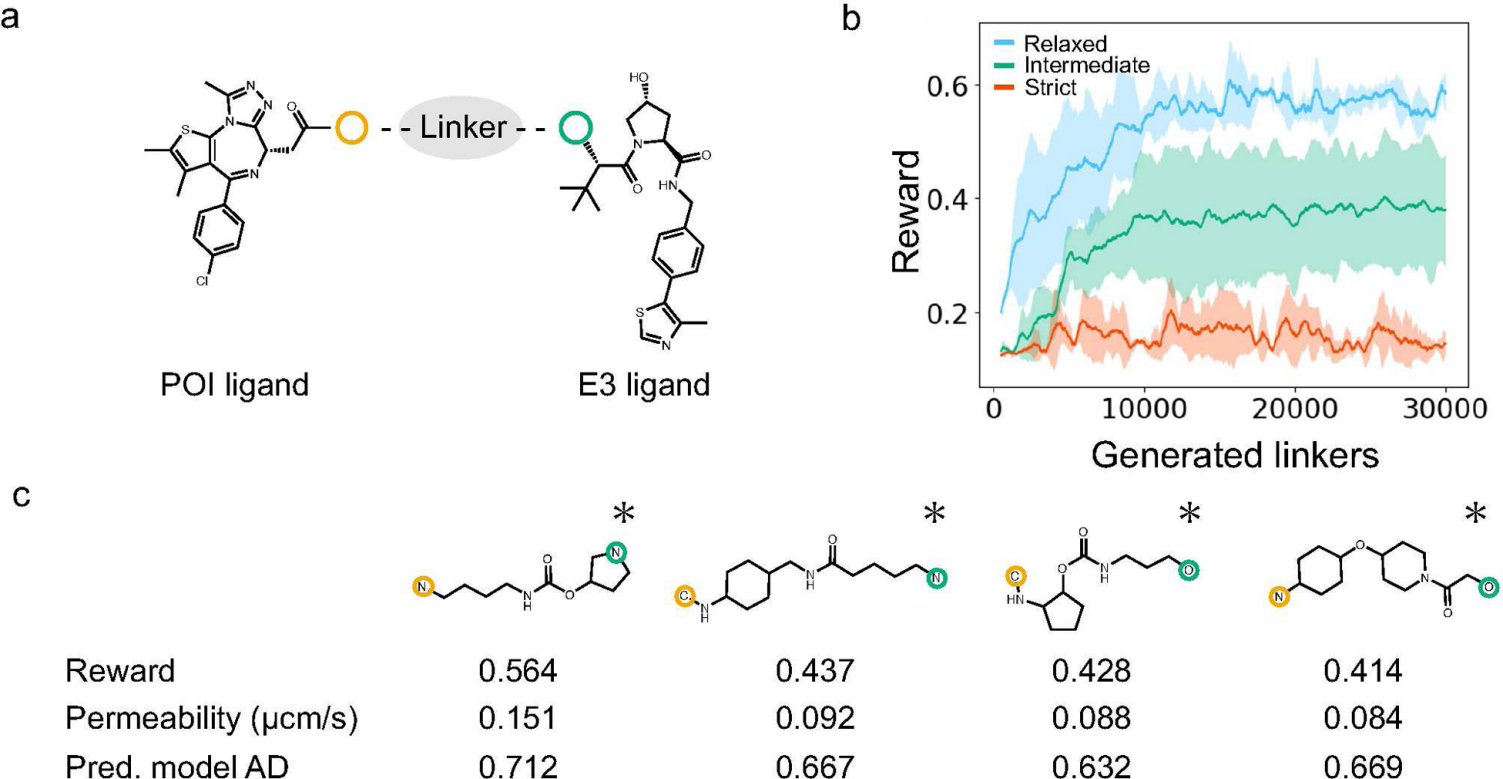
Results of linker design based on the BRD4 and VHL ligands. (a) Selected ligands. The yellow and green circles indicate the junction points with a linker. (b) Reward trends during linker generation. The blue, green, and red solid lines represent the moving average (window size = 500 linkers) of rewards from three independent runs under the relaxed, intermediate, and strict conditions, respectively. Shaded areas indicate the standard deviation across the runs. (c) Representative top-ranking linkers designed under the strict condition (described in Section 4.3). Atoms circled in yellow and green correspond to the junction points of the POI and E3 ligands, respectively. Asterisks denote PROTACs with linkers absent from PROTAC-DB 3.0.

#### 2.2.2 PROTAC Linker Design Based on BRD4 and Cereblon (CRBN) Ligands

PROTAC linkers were designed based on the BRD4 and CRBN ligands,^48^ as shown in Figure 4a, using PROTAC-TS. The PROTAC linkers were designed under relaxed, intermediate, and strict filtering conditions (Figures S5–S8). Under the relaxed condition, the reward values improved progressively during the design process, as shown in Figure 4b. Under the strict condition, PROTAC-TS successfully reproduced the linker of Compound 2895 ^48^, a PROTAC with high cell membrane permeability listed in PROTAC-DB 3.0, with a high reward value, as shown in Figure 4c. PROTAC-TS was also used to design other linkers for PROTACs that either achieved high reward values or were structurally similar to Compound 2895, as shown in Figure 4c,d. The experimental value of the cell membrane permeability of Compound 2895, as recorded in PROTAC-DB 3.0, was 2.14 μcm/s (log_10_ scale: 0.3304), whereas the predicted value was 0.91 μcm/s (log_10_ scale: −0.0402). The difference between the experimental and predicted values fell within the RMSE of the prediction model, suggesting that the prediction model retained a reasonable level of prediction performance, even for PROTACs not included in the training data set. These results indicated that PROTAC-TS enables the generation of linkers for PROTACs with high cell membrane permeability that are not present in the training data set and shows potential for designing novel and promising PROTAC linkers.

**Figure 4:**
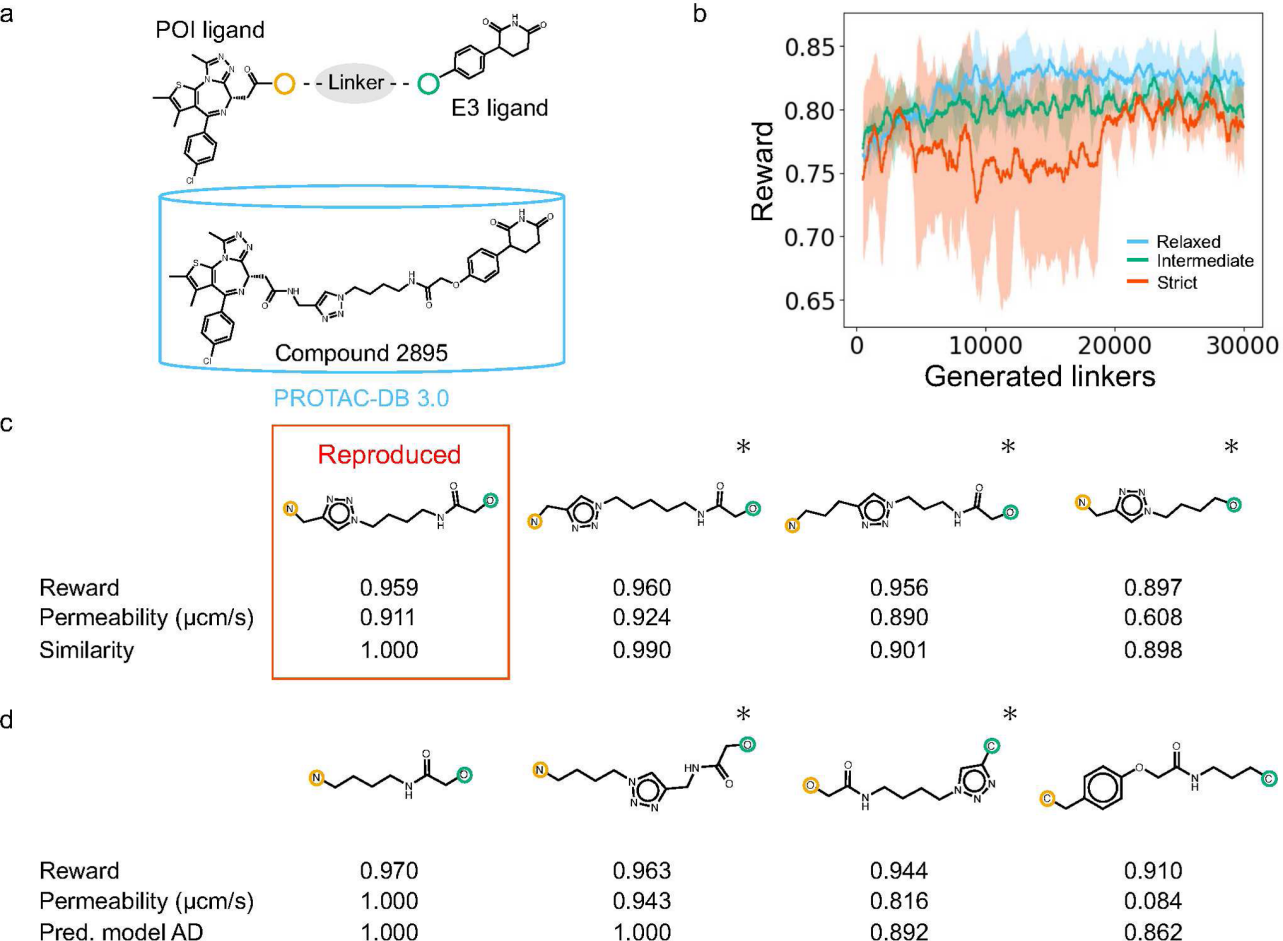
Results of linker design based on the BRD4 and CRBN ligands. (a) Selected ligands and Compound 2895. The yellow and green circles indicate the junction points with a linker. (b) Reward trends during linker generation. The blue, green, and red solid lines represent the moving average (window size = 500 linkers) of rewards from three independent runs under the relaxed, intermediate, and strict conditions, respectively. Shaded areas indicate the standard deviation across the runs. (c) The linker of Compound 2895 and linkers that showed high similarity to it, designed under the strict condition. Tanimoto similarity (denoted as “Similarity” in the figure) was calculated between Compound 2895 and each PROTAC with a designed linker using Morgan fingerprints, whose radius and dimension were 2 and 2,048, respectively. (d) Representative top-ranking linkers designed under the strict condition (described in Section 4.3). Atoms circled in yellow and green correspond to the junction points of the POI and E3 ligands, respectively. Asterisks denote PROTACs with linkers absent from PROTAC-DB 3.0.

### 2.3 Experimental Validation of PROTAC-TS

We applied PROTAC-TS to design linkers of PROTACs targeting interleukin-1 receptor-associated kinase 4 (IRAK4), Bruton’s tyrosine kinase (BTK), anaplastic lymphoma kinase (ALK), and BRD4. We selected five representative PROTACs from the designed PROTACs and experimentally evaluated their cell membrane permeability.

#### 2.3.1 IRAK4 PROTAC

Based on the IRAK4 and CRBN ligands, as shown in Figure 5a, PROTAC linkers were designed using PROTAC-TS under relaxed, intermediate, and strict filtering conditions (Figures S9–S12). Under the relaxed condition, reward values improved progressively during the design process, as shown in Figure 5b. Under the strict condition, PROTAC-TS successfully reproduced the linker of KT-474, excluding stereochemical information,^42^ as shown in Figure 5c. The experimental result for the cell membrane permeability of KT-474 is described in Section 2.3.5. In addition, linkers with higher reward values were designed, as shown in Figure 5d. Although the training data for the prediction model and the RNN-based linker generator did not include KT-474 and its linker, respectively, PROTAC-TS reproduced the KT-474 linker and designed multiple structurally similar linkers, as shown in Figure 5c, supporting the validity and generalizability of this method. The results presented herein highlight the potential of PROTAC-TS to design linkers for PROTACs with favorable cell membrane permeability comparable to those in clinical development and demonstrate the promise of PROTAC-TS as a valuable method for future PROTAC development.

**Figure 5:**
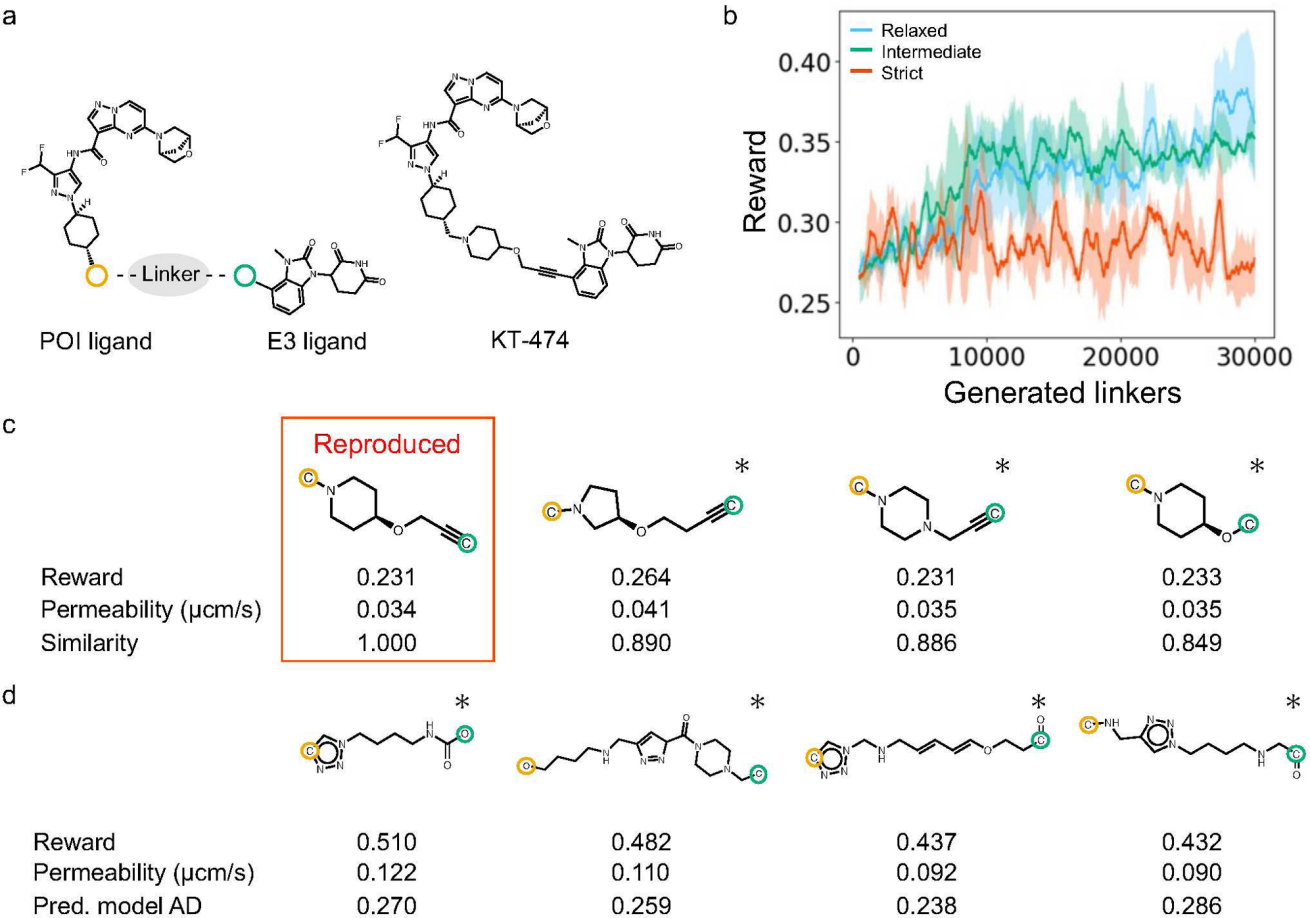
Results of linker design based on the IRAK4 and CRBN ligands. (a) Selected ligands and KT-474. The yellow and green circles indicate the junction points with a linker. (b) Reward trends during linker generation. The blue, green, and red solid lines represent the moving average (window size = 500 linkers) of rewards from three independent runs under the relaxed, intermediate, and strict conditions, respectively. Shaded areas indicate the standard deviation across the runs. (c) The linker of KT-474 and linkers that showed high similarity to it, designed under the strict condition. Tanimoto similarity (denoted as “Similarity” in the figure) was calculated between KT-474 and each PROTAC with a designed linker using Morgan fingerprints, whose radius and dimension were 2 and 2,048, respectively. (d) Representative top-ranking linkers designed under the strict condition (described in Section 4.3). Atoms circled in yellow and green correspond to the junction points of the POI and E3 ligands, respectively. Asterisks denote PROTACs with linkers absent from PROTAC-DB 3.0.

#### 2.3.2 BTK PROTAC

For the BTK and CRBN ligands, as shown in Figure 6a, PROTAC linkers were designed using PROTAC-TS under relaxed, intermediate, and strict filtering conditions (Figures S13 and S14). Under the intermediate condition, reward values improved progressively during the design process, as shown in Figure S14. This result indicates that PROTAC-TS can successfully design PROTAC linkers to improve predicted cell membrane permeability. In contrast, under the relaxed condition, reward values did not improve noticeably during the design process. Linker lengths remained unchanged, while the maximum branch length was relatively high during the design process, as shown in Figure S14. The limited improvement in reward is likely due to an insufficient exploration of longer linkers. This suggests that our method preferentially explored complex branching linkers rather than simple longer linkers under this condition. Under the strict condition, PROTAC-TS successfully reproduced the linker of NX-2127,^49^ which is currently undergoing phase I clinical trials. The experimental result for the cell membrane permeability of NX-2127 is described in Section 2.3.5.

**Figure 6:**
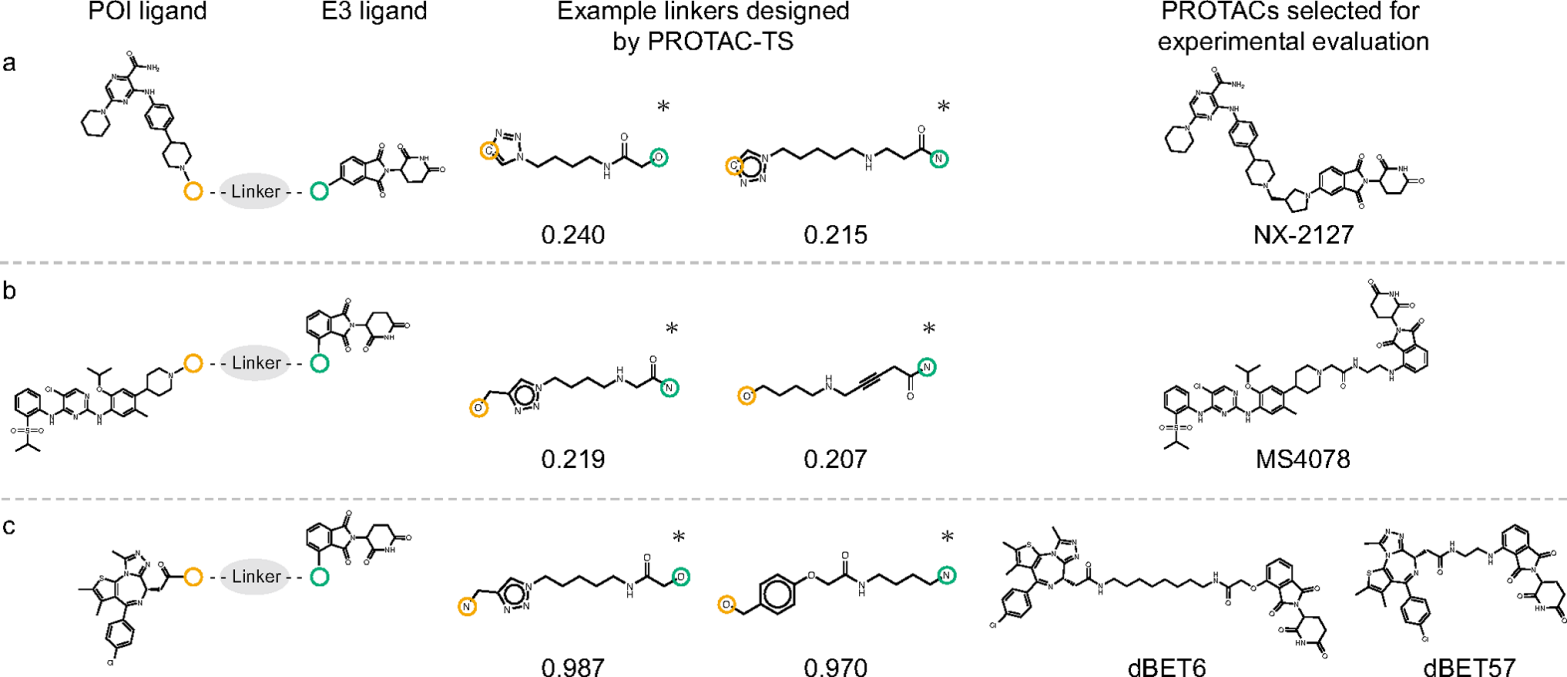
Results of linker design for PROTACs targeting (a) BTK, (b) ALK, and (c) BRD4, respectively. The left, middle, and right columns display the selected ligands, examples of the designed linkers under the strict condition, and PROTACs selected for experimental evaluation, respectively. Numerical values indicate reward scores. Atoms circled in yellow and green correspond to the junction points of the POI and E3 ligands, respectively. Asterisks denote PROTACs with linkers absent from PROTAC-DB 3.0.

#### 2.3.3 ALK PROTAC

PROTAC linkers for the ALK and CRBN ligands were designed using PROTAC-TS under relaxed, intermediate, and strict filtering conditions, as shown in Figure 6b. Similar to the results in Section 2.3.2, reward values improved progressively during the design process under the intermediate condition, whereas no noticeable improvement was observed under the relaxed and strict conditions, as shown in Figure S15. Under the relaxed condition, the limited improvement in reward values is likely attributable to an insufficient exploration of longer linkers. Under the strict condition, although the reward values did not show a marked upward trend, PROTAC-TS successfully designed chemically valid linker structures, as shown in Figure S16. In this study, we experimentally evaluated the cell membrane permeability of MS4078,^50^ which contains a linker generated under the strict condition and is commercially available, as described in Section 2.3.5.

#### 2.3.4 BRD4 PROTAC

For the BRD4 ligand and the thalidomide-based CRBN ligand, which is distinct from the one used in Section 2.2.2, as shown in Figure 6c, PROTAC linkers were designed using PROTAC-TS under relaxed, intermediate, and strict filtering conditions (Figures S17 and S18). Under both the relaxed and intermediate conditions, reward values improved during the early phase of the design process, as shown in Figure S18. In this study, we experimentally evaluated the cell membrane permeability of dBET6 ^51^ and dBET57,^52^ both of which contain linkers generated under the strict condition and are commercially available, as described in Section 2.3.5.

#### 2.3.5 Experimental Evaluation of Generated PROTACs

We experimentally evaluated the cell membrane permeability of KT-474, NX-2127, MS4078, dBET6, and dBET57. Details of the experimental conditions are described in Section 4.4. The experimental values are listed in Table S1. Overall, the experimental values tended to be higher than the predicted values. KT-474, dBET6, and dBET57 exhibited relatively high cell membrane permeability. For dBET6 and dBET57, the discrepancies between the predicted and experimental values were relatively small. Although NX-2127 and MS4078 showed comparatively large discrepancies, their experimental values fell within the 10th and 90th percentiles of the predictive distribution. Also, a moderate correlation was observed between the predicted and experimental values (R = 0.732), as shown in Figure S19. These results provide preliminary support for the robustness of the prediction model. In contrast, the experimental value of KT-474 fell outside this predictive percentile range. This range for KT-474 was wider than that for the other PROTACs. Additionally, the maximum Tanimoto similarity of KT-474 to any PROTAC in the training data set was lower than that of the other PROTACs, as shown in Table S2. These observations suggest that accurately estimating the cell membrane permeability of KT-474 is relatively challenging for the current prediction model.

## 3 Conclusions

In this study, we developed PROTAC-TS, a PROTAC linker design method for improving cell membrane permeability, by integrating a high-performance prediction model for cell membrane permeability. PROTAC-TS enables linker design while accounting for the AD of the prediction model to address the uncertainty stemming from the limited size of the training data set. PROTAC-TS successfully designed linkers of PROTACs with high predicted cell membrane permeability. Notably, this method reproduced the linker of Compound 2895, a PROTAC with high cell membrane permeability in PROTAC-DB 3.0, and the linkers of PROTACs that have entered clinical trials. PROTAC-TS further designed other linkers of PROTACs that were predicted to exhibit high cell membrane permeability. In addition, our results demonstrate that PROTAC linkers designed with high reward values tend to exhibit relatively high flexibility, although some contain ring structures. This trend suggests that PROTAC-TS has the potential to design PROTAC linkers while considering the chameleonicity,^53^ which is one possible way to improve cell membrane permeability by adapting molecular conformations in response to the surrounding environment. Furthermore, experimental evaluations confirmed that PROTACs containing linkers designed by PROTAC-TS, such as dBET6 and KT-474, exhibited relatively high cell membrane permeability. In PROTAC-TS, the reward function and filtering conditions can be modified as required. Also, PROTAC-TS has the potential to help medicinal chemists avoid overlooking promising candidates and to provide insights that may inform novel linker design strategies by exploring a diverse range of candidate molecules considering cell membrane permeability and PROTAC likeness. The results presented herein suggest that PROTAC-TS is a promising approach for accelerating the design of PROTACs with favorable cell membrane permeability.

Despite its reported advances, several aspects of this study require further improvement. The first challenge was the size of the data set used to train the prediction model for cell membrane permeability. Experimental data on the cell membrane permeability of PROTACs are scarce.^8^ While we addressed the limitation of the small data set using the prediction-model AD filter during linker design in this study, expansion of the data set will be desirable in future studies. The second challenge was an evaluation metric for cell membrane permeability. Although Caco-2 cell-based assays were employed in this study, other assay types such as parallel artificial membrane permeability assays are commonly used for cell membrane permeability assessments. PROTACs are often susceptible to the effects of efflux transporters,^54,55^ necessitating careful consideration of assay choice to ensure physiological relevance. The third challenge was the lack of consideration of properties beyond cell membrane permeability. PROTAC design requires balancing additional properties such as degradation activity toward POIs and solubility. Future studies should address these challenges to advance the development of a more comprehensive and practical PROTAC design platform.

## 4 Methods

### 4.1 Prediction Models for Cell Membrane Permeability

We evaluated four types of molecular features derived from the simplified molecular input line entry system (SMILES)^56^ and three prediction methods. The features included Mordred descriptors containing only 2D descriptors,^57^ Mordred descriptors containing 3D descriptors, bit-based Morgan fingerprints,^58,59^ and count-based Morgan fingerprints. For the 3D Mordred descriptors, the average descriptor values calculated from 10 conformers generated using RDKit software^60^ were used. Morgan fingerprints with a radius of 2 were also calculated using the RDKit software. TabPFN, LightGBM, and AutoGluon were evaluated as prediction models. TabPFN and AutoGluon were used with default parameters, whereas the hyperparameters of LightGBM were optimized using Optuna software.^61^ The experimentally measured A-to-B permeability across Caco-2 cell membranes was converted to a log_10_ scale and used as the target variable. The training data set consisted of 43 compounds with available Caco-2 permeability data from PROTAC-DB 3.0, with compound 258 excluded because of different experimental conditions. For data entries reported as “<*x* (μcm/s),” the value was replaced with “*x*/2 (μcm/s)” (e.g., <2 (μcm/s) was replaced with 1 (μcm/s)). In Section 2.2.2, the prediction model was reconstructed using the training data, excluding Compound 2895. The result of the prediction model is shown in Figure S20. Model performance was evaluated using leave-one-out cross-validation, with *R*, *R* ^2^, and RMSE as the evaluation metrics.

### 4.2 Linker Design Method

We developed PROTAC-TS by implementing a linker generation function in ChemTSv2, which is an ML-based de novo molecule generator. ChemTSv2 comprises RNN and MCTS algorithms based on reinforcement learning. The RNN-based linker generator was trained on the SMILES representations of the linkers and generated new SMILES strings. MCTS explores the chemical space based on a predefined reward function to design molecules with desirable properties. The RNN-based linker generator was modified to treat an asterisk as an attachment point between the linker and ligands. For training, 2749 SMILES linkers were curated from PROTAC-DB 3.0 by removing the ionized structures from the original set of 2753 SMILES linkers. These linkers were randomized four times and added to the curated data set. After removing duplicates, 12,863 SMILES linkers were used as the training data. For the RNN-based linker generation described in Sections 2.2.2, 2.3.2, 2.3.3, and 2.3.4, the linkers of Compound 2895, NX-2127, MS4078, and those of dBET6 and dBET57 were excluded from the training data set, respectively. The reward function was defined as a left-sided Gaussian function with parameters μ = 0.25 and σ = 1, based on predicted cell membrane permeability values, as shown in Figure S21.

PROTAC-TS supports the application of filters for generating linkers. In this study, linkers were designed under three filtering conditions (relaxed, intermediate, and strict), as shown in Table S3. The relaxed condition consisted of attachment-point, linker-validation, and radical-atom filters. The attachment-point filter excluded the SMILES linker in which the number of asterisks used to indicate the points of connection to a POI ligand and an E3 ligand was not equal to two. The linker-validation filter excluded linkers that resulted in invalid molecules when attached to both ligands. The radical-atom filter excluded linkers that contained radical electrons. The intermediate condition consisted of filters applied to each linker and the corresponding PROTAC generated by attaching the linker to both ligands. The filters applied to the linkers included attachment-point, linker-validation, radical-atom, ring-structure, and branched-structure filters. The ring-structure filter excluded linkers containing ring substructures with fewer than five or more than six members. The branched-structure filter excludes linkers containing two or more consecutive atoms branching from the shortest path between two attachment points. Atoms within a ring structure were not considered branches if the ring itself was part of the shortest path. The filters applied to PROTACs included alert-substructure and specific-substructure filters. The alert-substructure filter excluded linkers that resulted in PROTACs containing substructures listed under “Common Alerts” in the medchem package.^62^ In Section 2.3.4, the alert-substructure filter was not applied. The specific substructure filter excluded linkers that resulted in PROTACs containing substructures that were considered synthetically challenging or chemically unstable, as specified in the SMILES arbitrary target specification format, as shown in Table S4. The strict condition also consisted of filters applied to each linker and the corresponding PROTAC generated by attaching the linker to both ligands. The filters applied to the linkers include attachment-point, linker-validation, radical-atom, ring-structure, branched-structure, linker-length, and linker-similarity filters. The linker-length filter excluded linkers whose shortest path length between attachment points exceeded 15. The linker-similarity filter excluded linkers whose maximum Tanimoto similarity to any of the 2748 linkers used to train the RNN-based linker generator was below 0.3. The filters applied to the PROTACs included alert-substructure, specific-substructure, and prediction-model-AD filters. The prediction-model-AD filter excluded linkers that resulted in PROTACs falling outside the AD of the prediction model. To define AD in this study, we adopted the maximum value of the Tanimoto similarity for the training data, which is a simple and commonly used method.^41,63–65^ The similarity was calculated using Morgan fingerprints with radii and dimensions of 2 and 2,048, respectively. The threshold was set to 0.1. For each condition, 30,000 linkers were designed for each of three independent runs. To reproduce the linkers of Compound 2895 and KT-474, 30,000 linkers were designed in 10 and 100 independent runs, respectively.

### 4.3 Linker Clustering

Clustering analysis was performed to visualize the structural diversity of the designed linkers. The analysis was applied to PROTAC linkers designed under all filtering conditions for Sections 2.2.1, 2.2.2, and 2.3.1, and the strict conditions for Sections 2.3.2, 2.3.3, and 2.3.4. The top 5000 generated linkers, ranked by reward values, were clustered using the K-medoids algorithm with Morgan fingerprints as input features. The Morgan fingerprints were computed with a radius of 2 and a dimensionality of 2048. The number of clusters was set to 10.

### 4.4 Caco-2 Cell Membrane Permeability Study

KT-474 (Catalog No. E1655), NX-2127 (Catalog No. E1381), MS4078 (Catalog No. S0072), dBET6 (Catalog No. S8762), and dBET57 (Catalog No. S0137) were purchased from Selleck Chemicals (Houston, TX, USA). For this evaluation, the S-enantiomer of NX-2127, which possesses a stereogenic center at the pyrrolidine moiety, was used. The Caco-2 cell permeability assay using these PROTACs was outsourced to Axcelead Drug Discovery Partners, Inc. (Kanagawa, Japan). Caco-2 cells (American Type Culture Collection, Manassas, VA, USA) were cultured, and a transcellular transport study was performed. The cells were cultured in Transwell 96-well permeable support (pore size 0.4 μm, 0.11 cm^2^ surface area) with a polycarbonate membrane (PSHT004S5, Millipore Corporation, Bedford, MA, USA). The cells were preincubated with bovine serum albumin (BSA)-free Hank’s balanced salt solution (HBSS) in the apical compartment (75 μL) and HBSS containing 4% BSA in the basal compartment (250 μL) for 10 min at 37 °C. Subsequently, transcellular transport was initiated by the addition of BSA-free HBSS to apical compartments containing 10 μM compounds. The assay was terminated by separating each assay plate after 2 h. Aliquots (50 μL) from the basolateral compartment were mixed with acetonitrile/methanol. After centrifugation, the compound concentrations in the supernatant were measured using liquid chromatography-tandem mass spectrometry (LC–MS/MS) and a Kinetex C18 column (2.6 μm, 2.1 × 30 mm). The apparent permeabilities (*P*_app_, AtoB) of the receivers were determined. Lucifer yellow was added onto the apical compartment at a concentration of 100 μM. The *P*_app_ of lucifer yellow in the wells where each test compound was evaluated was reported. The *P*_app_ value of each compound in the membrane permeability test was calculated from a one-point standard curve (0.1 μM) using the following equation.

$$P_{app}=\frac{C\times V}{T\times A\times C_{0}}$$

where *C* (μM) is the concentration of the test compound on the basal side after incubation (measured value); *V* (mL) is the volume of the basolateral compartment (0.25 mL); *T* (s) is the permeation experiment time (7200 s); *A* (cm^2^) is the membrane area (0.11 cm^2^); and C_0_ (μM) is the concentration of test compound (measured value).

## Supplementary Material



## Data Availability

The source codes and models are available via https://github.com/ycu-iil/PROTAC-TS.
