# Supplementary material for: Data-Driven Design of PROTAC Linkers to Improve PROTAC Cell Membrane Permeability
Source: JACS Au. 2026 Feb 9;6(2):1400–10. doi: 10.1021/jacsau.6c00033 (PMC12933330; doi:10.1021/jacsau.6c00033)
Supplement: Supplementary file 1 [file au6c00033_si_001.pdf]

Electronic Supplementary Information

Data-Driven Design of PROTAC Linkers to Improve PROTAC Cell Membrane Permeability

Yuki Murakami [1

Shoichi Ishida [1

Nobuo Cho [1

Hitomi Yuki [2

Masateru Ohta [3

Teruki Honma [2

Yosuke Demizu [1, 4

Kei Terayama [1, 5, 6

1. Graduate School of Medical Life Science, Yokohama City University, 1-7-29, Suehiro-cho, Tsurumi-ku, Yokohama, Kanagawa 230-0045, Japan.
2. RIKEN Center for Integrative Medical Sciences, 1-7-22, Suehiro-cho, Tsurumi-ku, Yokohama Kanagawa 230-0045, Japan.
3. HPC- and AI-driven Drug Development Platform Division, RIKEN Center for Computational Science, 1-7-22, Suehiro-cho, Tsurumi-ku, Yokohama, Kanagawa 230-0045, Japan.
4. Division of Organic Chemistry, National Institute of Health Sciences, 3-25-26, Tonomachi, Kawasaki-ku, Kawasaki, Kanagawa 210-9501, Japan.
5. RIKEN Center for Advanced Intelligence Project, 1-4-1, Nihonbashi, Chuo-ku, Tokyo 103-0027, Japan.
6. MDX Research Center for Element Strategy, Institute of Science Tokyo, 4259, Nagatsuta-cho, Midori-ku, Yokohama, Kanagawa 226-8501, Japan.

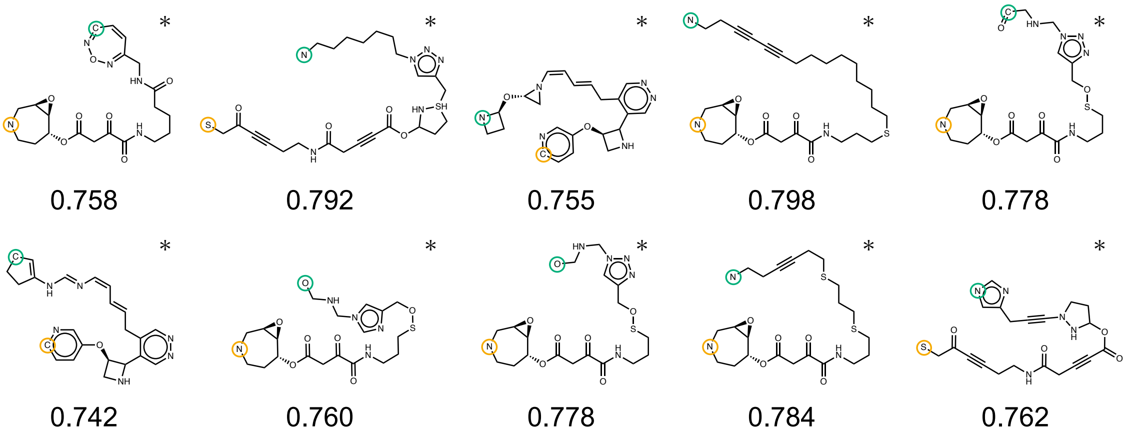

Figure S1. Examples of linkers designed based on the BRD4 and VHL ligands under the relaxed condition in Section 2.2.1. The displayed linkers correspond to the PROTACs with the top reward scores within each cluster (Section 4.3). The values represent reward scores. Atoms circled in yellow and green correspond to the junction points of the POI and E3 ligands, respectively. Asterisks denote PROTACs with linkers absent from PROTAC-DB 3.0.

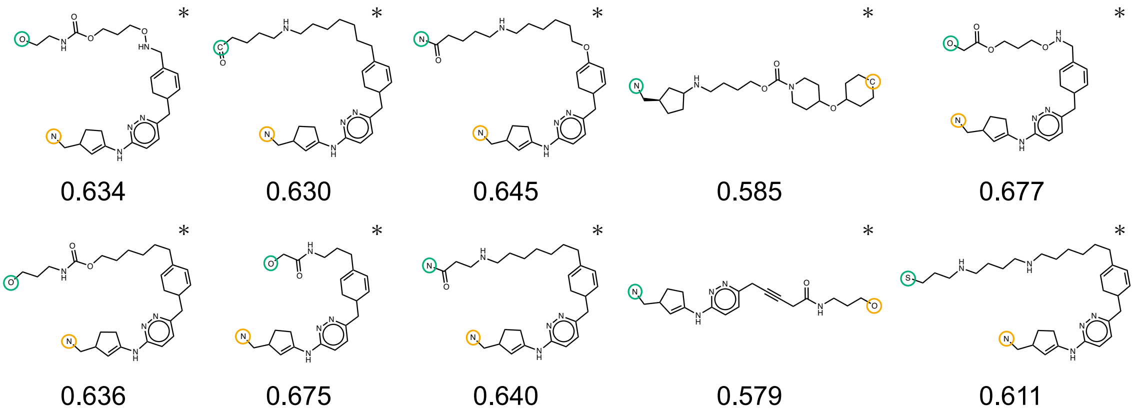

Figure S2. Examples of linkers designed based on the BRD4 and VHL ligands under the intermediate condition in Section 2.2.1. The displayed linkers correspond to the PROTACs with the top reward scores within each cluster (Section 4.3). The values represent reward scores. Atoms circled in yellow and green correspond to the junction points of the POI and E3 ligands, respectively. Asterisks denote PROTACs with linkers absent from PROTAC-DB 3.0.

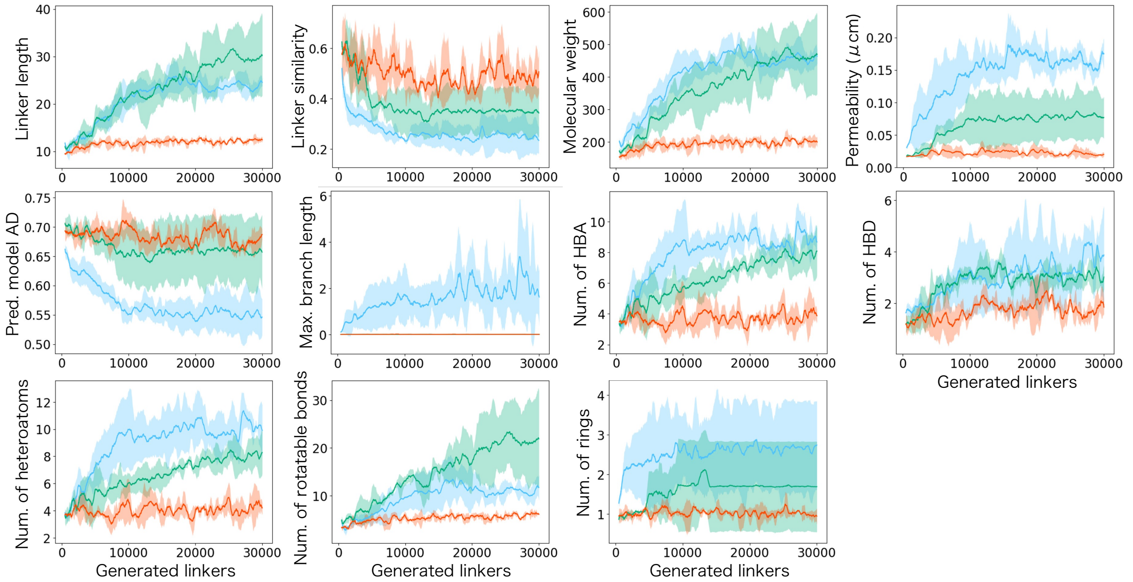

Figure S3. Trends in molecular properties during the linker generation process based on the BRD4 and VHL ligands in Section 2.2.1. Molecular weight, the number of hydrogen bond acceptors (HBAs), the number of hydrogen bond donors (HBDs), the number of heteroatoms, the number of rotatable bonds, and the number of rings were all calculated using RDKit software. For these calculations, linker structures were modified by capping ligand attachment points with hydrogen atoms. Linker length represents the shortest path length between the attachment points. Linker similarity represents the maximum Tanimoto similarity to any of the 2748 linkers used to train the RNN-based linker generator. Prediction model AD represents the maximum Tanimoto similarity to any molecule in the training data of the prediction model for cell membrane permeability. These similarities were calculated using Morgan fingerprints, whose radius and dimension were 2 and 2048, respectively. Maximum branch length represents the maximum number of consecutive atoms branching from the shortest path between the attachment points, where atoms within a ring structure are not considered branches if the ring itself is part of the shortest path. The blue, green, and red solid lines represent the moving average (window size = 500 linkers) of rewards from three independent runs under the relaxed, intermediate, and strict conditions, respectively.

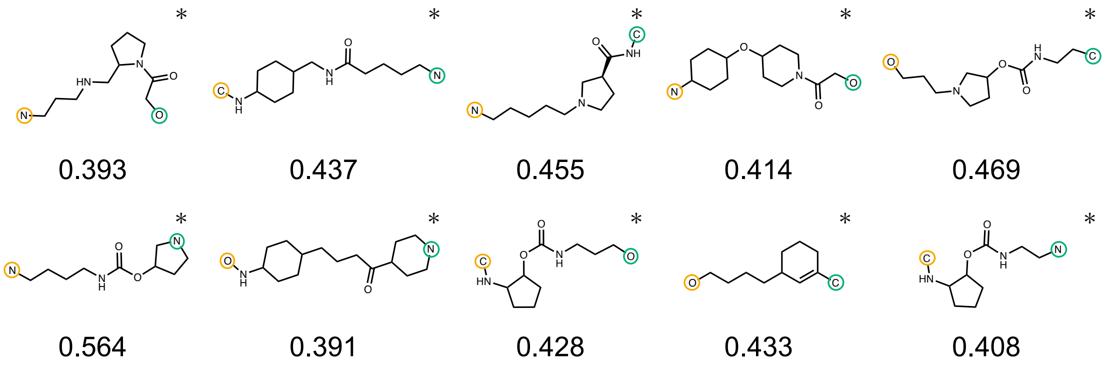

Figure S4. Examples of linkers designed based on the BRD4 and VHL ligands under the strict condition in Section 2.2.1. The displayed linkers correspond to the PROTACs with the top reward scores within each cluster (Section 4.3). The values represent reward scores. Atoms circled in yellow and green correspond to the junction points of the POI and E3 ligands, respectively. Asterisks denote PROTACs with linkers absent from PROTAC-DB 3.0.

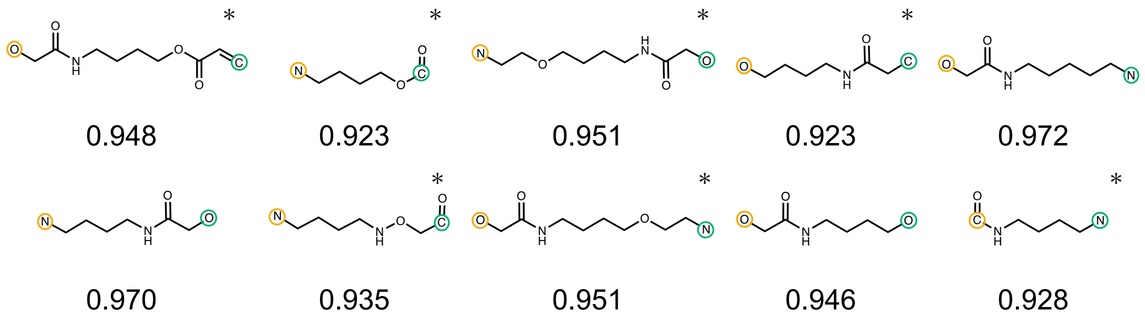

Figure S5. Examples of linkers designed based on the BRD4 and CRBN ligands under the relaxed condition in Section 2.2.2. The displayed linkers correspond to the PROTACs with the top reward scores within each cluster (Section 4.3). The values represent reward scores. Atoms circled in yellow and green correspond to the junction points of the POI and E3 ligands, respectively. Asterisks denote PROTACs with linkers absent from PROTAC-DB 3.0.

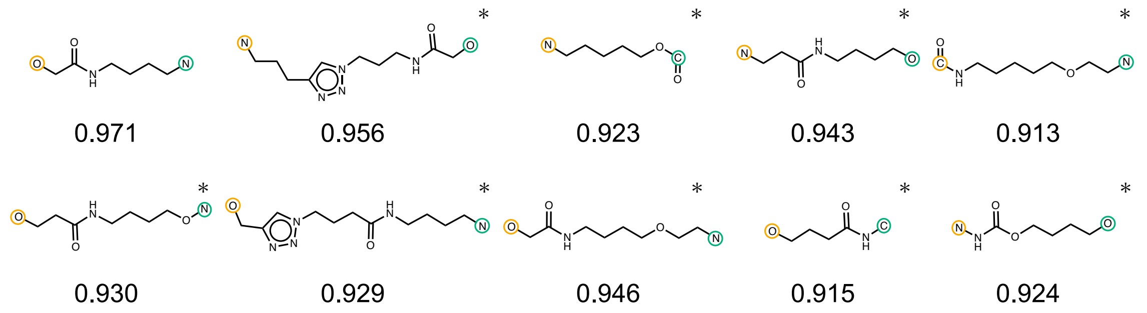

Figure S6. Examples of linkers designed based on the BRD4 and CRBN ligands under the intermediate condition in Section 2.2.2. The displayed linkers correspond to the PROTACs with the top reward scores within each cluster (Section 4.3). The values represent reward scores. Atoms circled in yellow and green correspond to the junction points of the POI and E3 ligands, respectively. Asterisks denote PROTACs with linkers absent from PROTAC-DB 3.0.

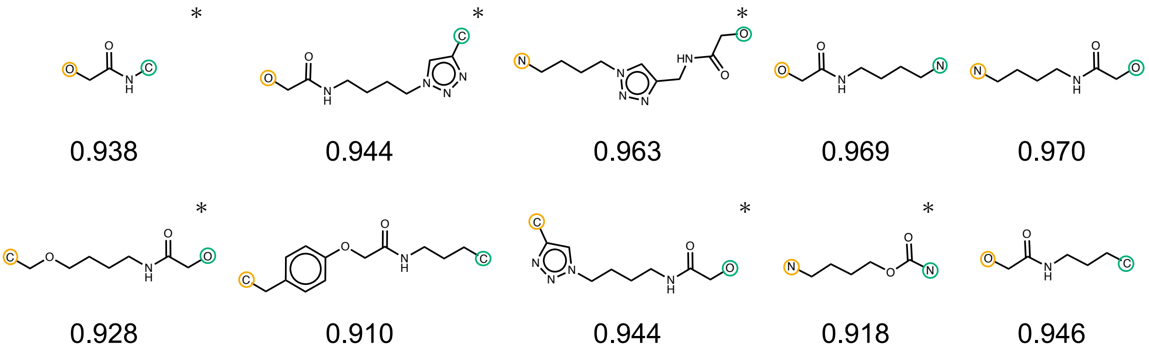

Figure S7. Examples of linkers designed based on the BRD4 and CRBN ligands under the strict condition in Section 2.2.2. The displayed linkers correspond to the PROTACs with the top reward scores within each cluster (Section 4.3). The values represent reward scores. Atoms circled in yellow and green correspond to the junction points of the POI and E3 ligands, respectively. Asterisks denote PROTACs with linkers absent from PROTAC-DB 3.0.

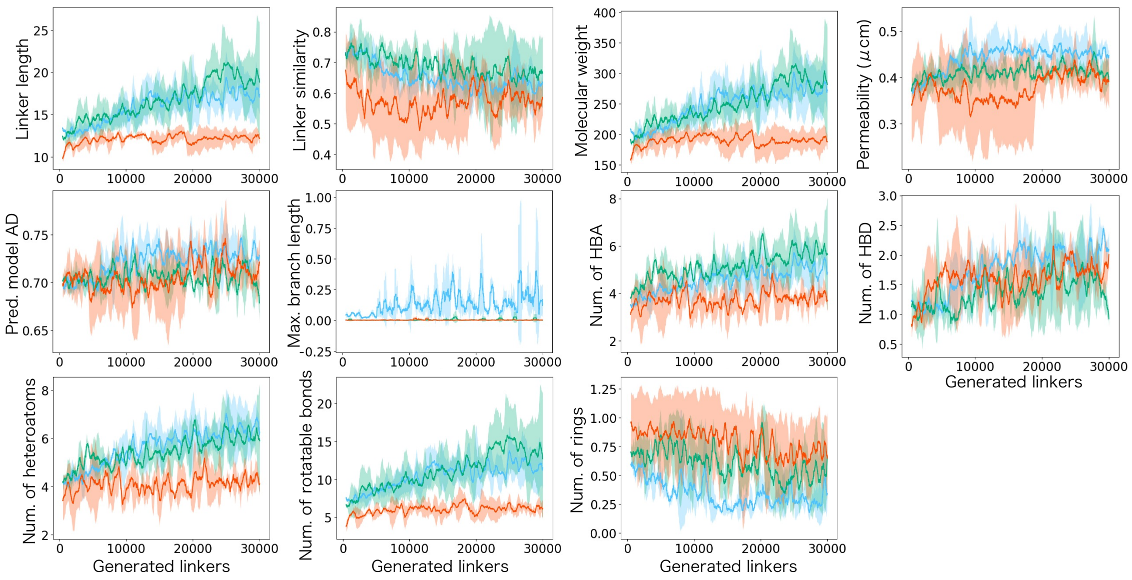

Figure S8. Trends in molecular properties during the linker generation process based on the BRD4 and CRBN ligands in Section 2.2.2. Molecular weight, the number of HBAs, the number of HBDs, the number of heteroatoms, the number of rotatable bonds, and the number of rings were all calculated using RDKit software. For these calculations, linker structures were modified by capping ligand attachment points with hydrogen atoms. Linker length represents the shortest path length between the attachment points. Linker similarity represents the maximum Tanimoto similarity to any of the 2748 linkers used to train the RNN-based linker generator. Prediction model AD represents the maximum Tanimoto similarity to any molecule in the training data of the prediction model for cell membrane permeability. These similarities were calculated using Morgan fingerprints, whose radius and dimension were 2 and 2048, respectively. Maximum branch length represents the maximum number of consecutive atoms branching from the shortest path between the attachment points, where atoms within a ring structure are not considered branches if the ring itself is part of the shortest path. The blue, green, and red solid lines represent the moving average (window size = 500 linkers) of rewards from three independent runs under the relaxed, intermediate, and strict conditions, respectively.

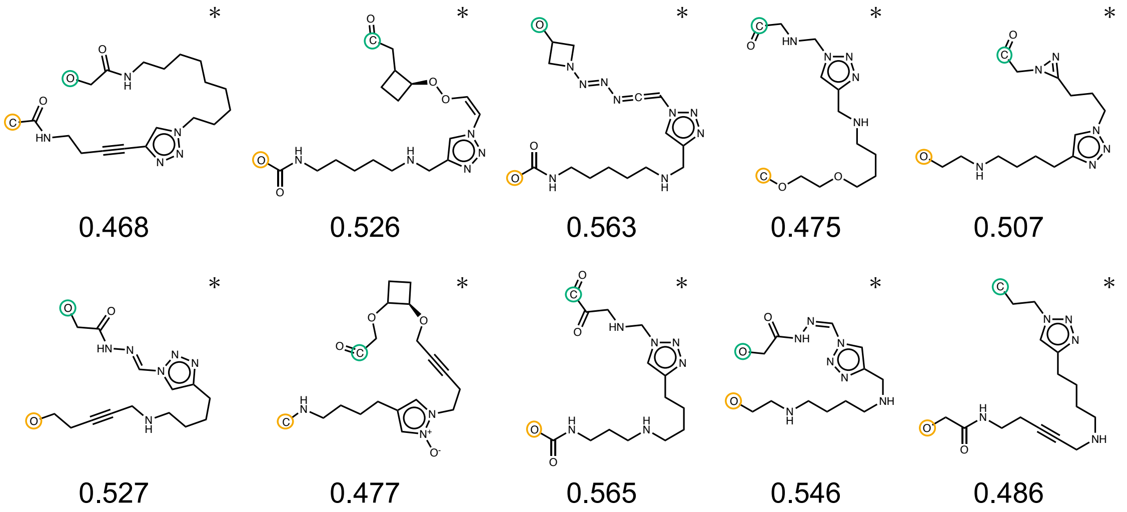

Figure S9. Examples of linkers designed based on the IRAK4 and CRBN ligands under the relaxed condition in Section 2.3.1. The displayed linkers correspond to the PROTACs with the top reward scores within each cluster (Section 4.3). The values represent reward scores. Atoms circled in yellow and green correspond to the junction points of the POI and E3 ligands, respectively. Asterisks denote PROTACs with linkers absent from PROTAC-DB 3.0.

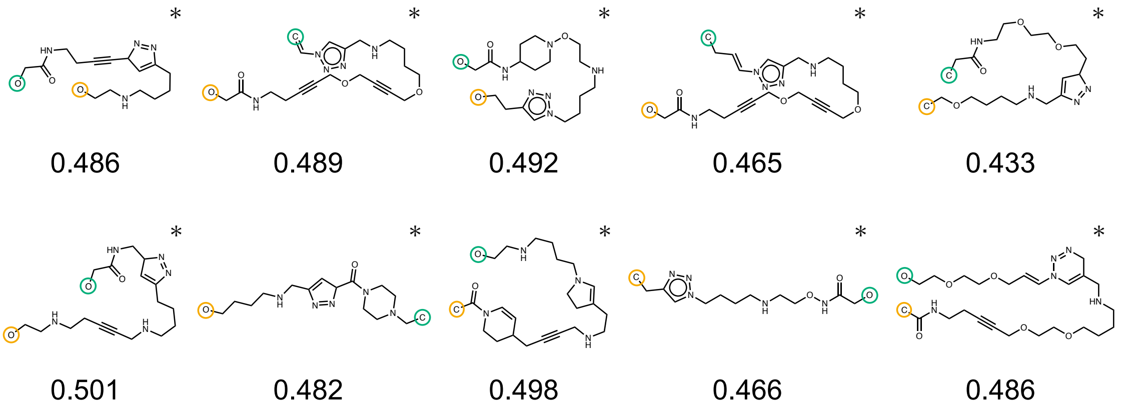

Figure S10. Examples of linkers designed based on the IRAK4 and CRBN ligands under the intermediate condition in Section 2.3.1. The displayed linkers correspond to the PROTACs with the top reward scores within each cluster (Section 4.3). The values represent reward scores. Atoms circled in yellow and green correspond to the junction points of the POI and E3 ligands, respectively. Asterisks denote PROTACs with linkers absent from PROTAC-DB 3.0.

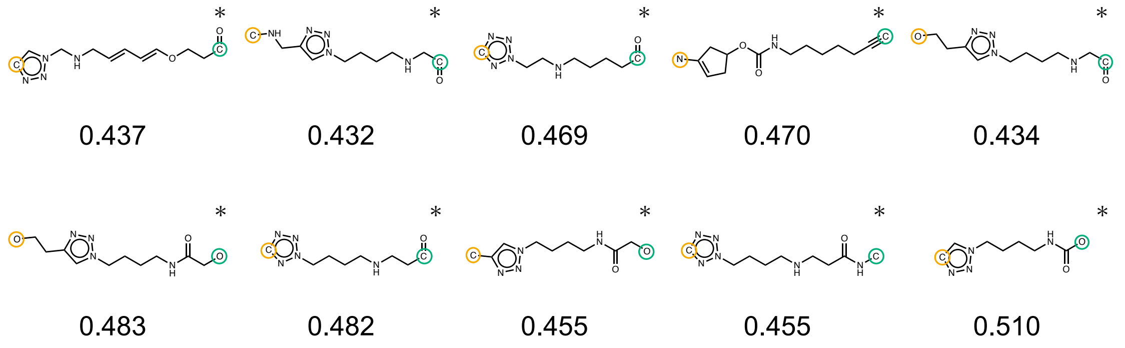

Figure S11. Examples of linkers designed based on the IRAK4 and CRBN ligands under the strict condition in Section 2.3.1. The displayed linkers correspond to the PROTACs with the top reward scores within each cluster (Section 4.3). The values represent reward scores. Atoms circled in yellow and green correspond to the junction points of the POI and E3 ligands, respectively. Asterisks denote PROTACs with linkers absent from PROTAC-DB 3.0.

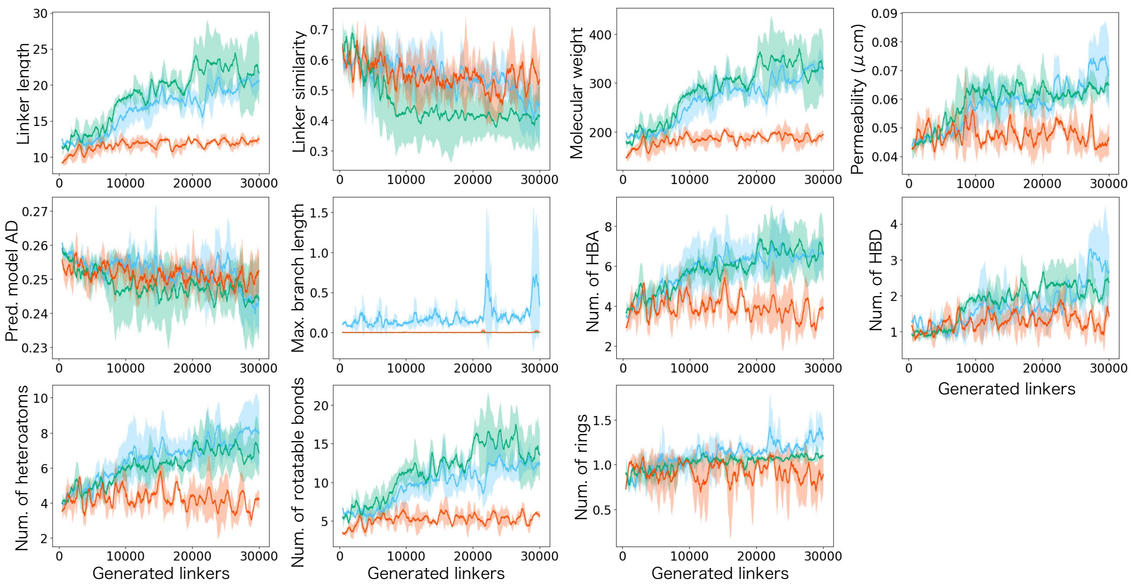

Figure S12. Trends in molecular properties during the linker generation process based on the IRAK4 and CRBN ligands in Section 2.3.1. Molecular weight, the number of HBAs, the number of HBDs, the number of heteroatoms, the number of rotatable bonds, and the number of rings were all calculated using RDKit software. For these calculations, linker structures were modified by capping ligand attachment points with hydrogen atoms. Linker length represents the shortest path length between attachment points. Linker similarity represents the maximum Tanimoto similarity to any of the 2748 linkers used to train the RNN-based linker generator. Prediction model AD represents the maximum Tanimoto similarity to any molecule in the training data of the prediction model for cell membrane permeability. These similarities were calculated using Morgan fingerprints, whose radius and dimension were 2 and 2048, respectively. Maximum branch length represents the maximum number of consecutive atoms branching from the shortest path between the attachment points, where atoms within a ring structure are not considered branches if the ring itself is part of the shortest path. The blue, green, and red solid lines represent the moving average (window size = 500 linkers) of rewards from three independent runs under the relaxed, intermediate, and strict conditions, respectively.

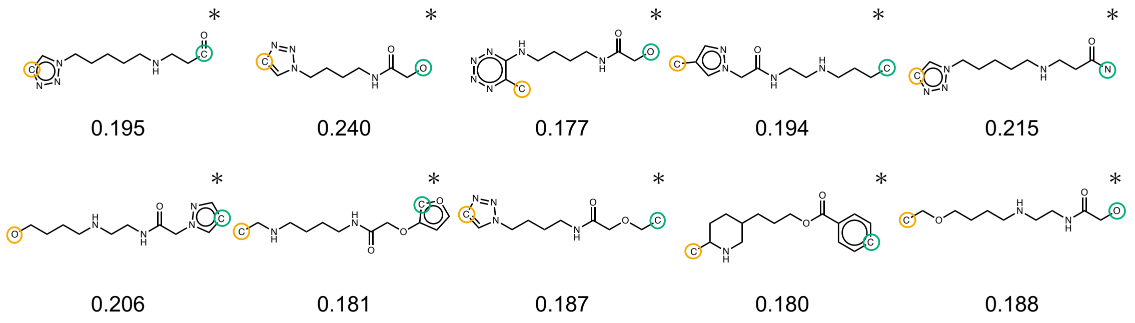

Figure S13. Examples of linkers designed based on the BTK and CRBN ligands under the strict condition in Section 2.3.2. The displayed linkers correspond to the PROTACs with the top reward scores within each cluster (Section 4.3). The values represent reward scores. Atoms circled in yellow and green correspond to the junction points of the POI and E3 ligands, respectively. Asterisks denote PROTACs with linkers absent from PROTAC-DB 3.0.

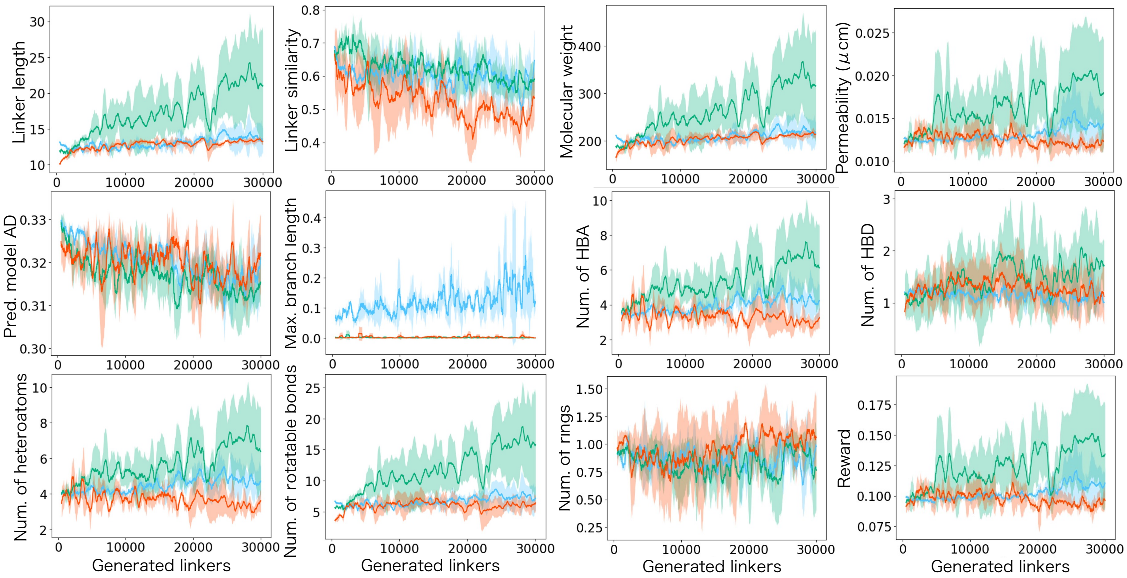

Figure S14 Trends in molecular properties during the linker generation process based on the BTK and CRBN ligands in Section 2.3.2. Molecular weight, the number of HBAs, the number of HBDs, the number of heteroatoms, the number of rotatable bonds, and the number of rings were all calculated using RDKit software. For these calculations, linker structures were modified by capping ligand attachment points with hydrogen atoms. Linker length represents the shortest path length between attachment points. Linker similarity represents the maximum Tanimoto similarity to any of the 2748 linkers used to train the RNN-based linker generator. Prediction model AD represents the maximum Tanimoto similarity to any molecule in the training data of the prediction model for cell membrane permeability. These similarities were calculated using Morgan fingerprints, whose radius and dimension were 2 and 2048, respectively. Maximum branch length represents the maximum number of consecutive atoms branching from the shortest path between the attachment points, where atoms within a ring structure are not considered branches if the ring itself is part of the shortest path. The blue, green, and red solid lines represent the moving average (window size = 500 linkers) of rewards from three independent runs under the relaxed, intermediate, and strict conditions, respectively.

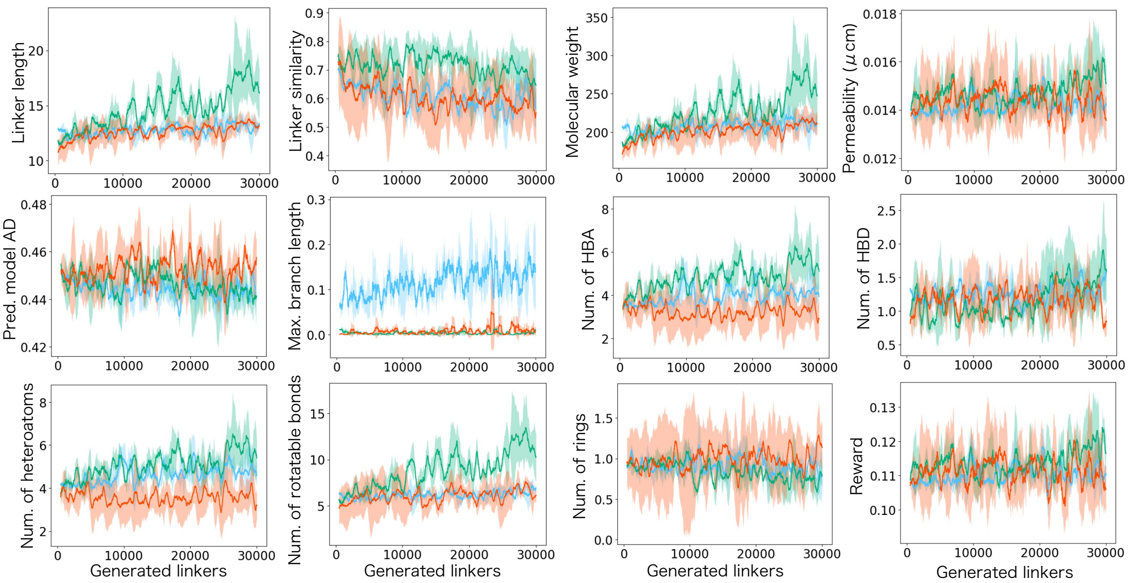

Figure S15. Trends in molecular properties during the linker generation process based on the ALK and CRBN ligands in Section 2.3.3. Molecular weight, the number of HBAs, the number of HBDs, the number of heteroatoms, the number of rotatable bonds, and the number of rings were all calculated using RDKit software. For these calculations, linker structures were modified by capping ligand attachment points with hydrogen atoms. Linker length represents the shortest path length between attachment points. Linker similarity represents the maximum Tanimoto similarity to any of the 2748 linkers used to train the RNN-based linker generator. Prediction model AD represents the maximum Tanimoto similarity to any molecule in the training data of the prediction model for cell membrane permeability. These similarities were calculated using Morgan fingerprints, whose radius and dimension were 2 and 2048, respectively. Maximum branch length represents the maximum number of consecutive atoms branching from the shortest path between the attachment points, where atoms within a ring structure are not considered branches if the ring itself is part of the shortest path. The blue, green, and red solid lines represent the moving average (window size = 500 linkers) of rewards from three independent runs under the relaxed, intermediate, and strict conditions, respectively.

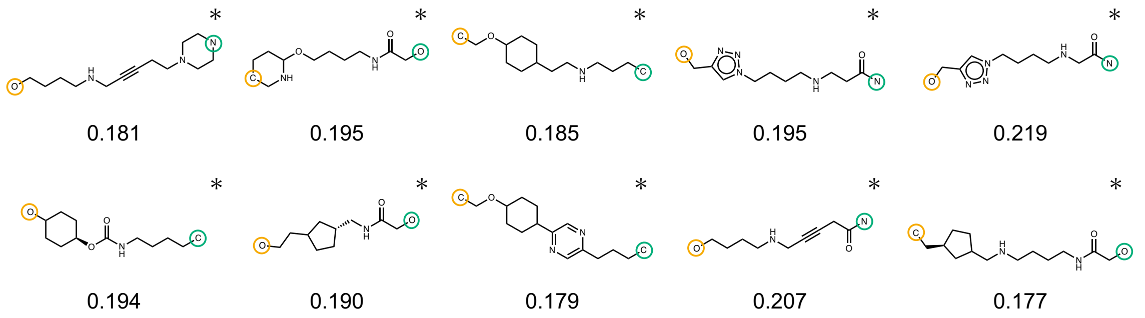

Figure S16. Examples of linkers designed based on the ALK and CRBN ligands under the strict condition in Section 2.3.3. The displayed linkers correspond to the PROTACs with the top reward scores within each cluster (Section 4.3). The values represent reward scores. Atoms circled in yellow and green correspond to the junction points of the POI and E3 ligands, respectively. Asterisks denote PROTACs with linkers absent from PROTAC-DB 3.0.

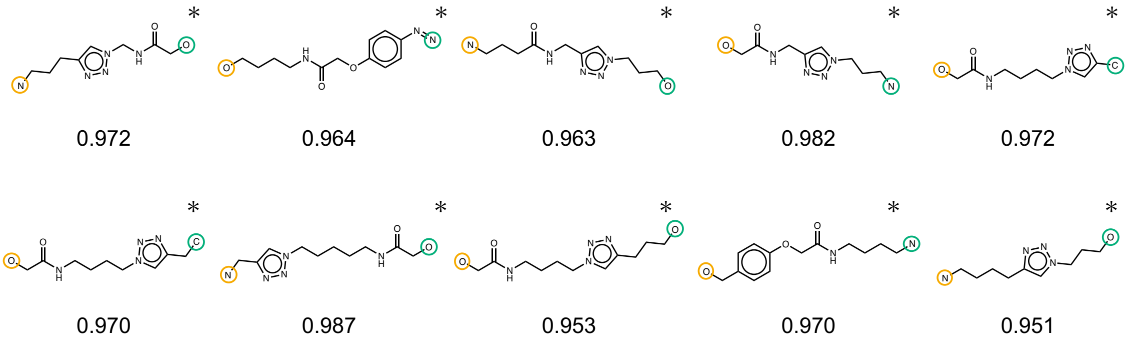

Figure S17. Examples of linkers designed based on the BRD4 and CRBN ligands under the strict condition in Section 2.3.4. The displayed linkers correspond to the PROTACs with the top reward scores within each cluster (Section 4.3). The values represent reward scores. Atoms circled in yellow and green correspond to the junction points of the POI and E3 ligands, respectively. Asterisks denote PROTACs with linkers absent from PROTAC-DB 3.0.

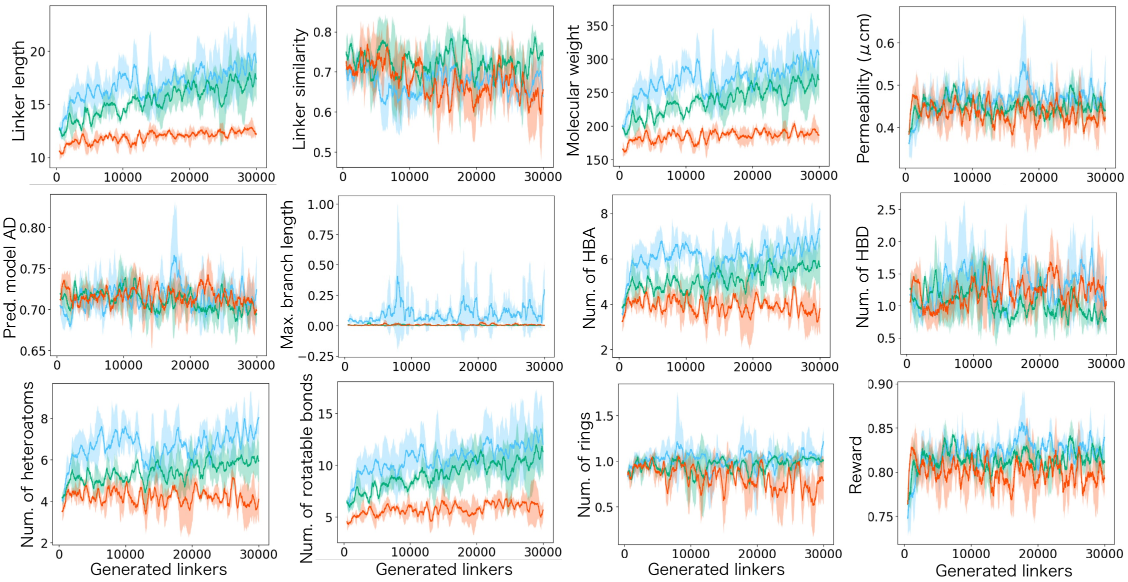

Figure S18. Trends in molecular properties during the linker generation process based on the BRD4 and CRBN ligands in Section 2.3.3. Molecular weight, the number of HBAs, the number of HBDs, the number of heteroatoms, the number of rotatable bonds, and the number of rings were all calculated using RDKit software. For these calculations, linker structures were modified by capping ligand attachment points with hydrogen atoms. Linker length represents the shortest path length between attachment points. Linker similarity represents the maximum Tanimoto similarity to any of the 2748 linkers used to train the RNN-based linker generator. Prediction model AD represents the maximum Tanimoto similarity to any molecule in the training data of the prediction model for cell membrane permeability. These similarities were calculated using Morgan fingerprints, whose radius and dimension were 2 and 2048, respectively. Maximum branch length represents the maximum number of consecutive atoms branching from the shortest path between the attachment points, where atoms within a ring structure are not considered branches if the ring itself is part of the shortest path. The blue, green, and red solid lines represent the moving average (window size = 500 linkers) of rewards from three independent runs under the relaxed, intermediate, and strict conditions, respectively.

Table S1. Experimental results for cell membrane permeability (μ cm/s; log10-transformed values in parentheses)

Section	Target	PROTAC	Ex. value	Pred. mean (log_{10} scale)	10th percentile (log_{10} scale)	90th percentile (log_{10} scale)
2.3.1.	IRAK4	KT-474	1.26 (0.100)	-1.45	-2.56	-0.26
2.3.2.	BTK	NX-2127	0.11 (-0.959)	-2.00	-2.71	-1.04
2.3.3.	ALK	MS4078	0.06 (-1.222)	-1.96	-2.79	-1.00
2.3.4.	BRD4	dBET6	0.99 (-0.004)	-0.02	-0.65	0.37
2.3.4.	BRD4	dBET57	0.52 (-0.284)	-0.78	-1.53	-0.10

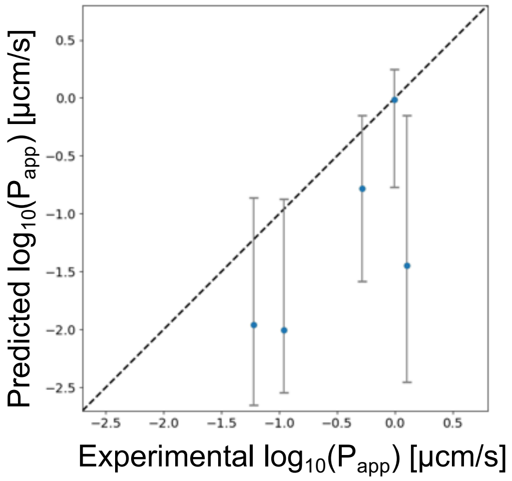

Figure S19. Scatterplot displaying the correlation between predicted and experimental cell membrane permeability of the experimentally evaluated PROTACs. Data points represent the mean of the predictive distribution generated by the prediction model. The upper and lower error bars denote the 90th and 10th percentiles of this distribution, respectively.

Table S2. Maximum Tanimoto similarity of the experimentally evaluated PROTACs to compounds in the training data set for the cell membrane permeability prediction model. The similarity was calculated using Morgan fingerprints with a radius of 2 and 2048 bits.

PROTAC	Maximum Tanimoto Similarity
KT-474	0.25
NX-2127	0.32
MS4078	0.47
dBET6	0.99
dBET57	0.80

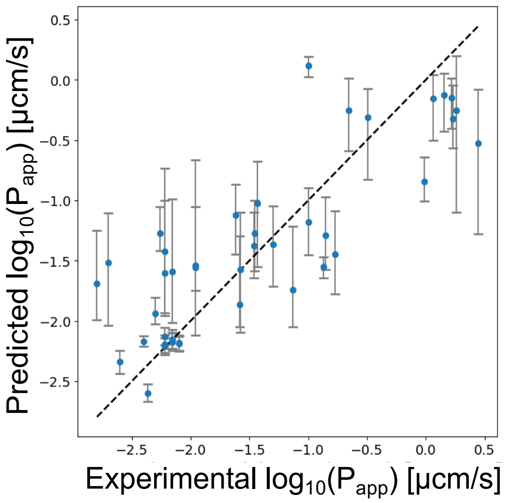

Figure S20. Scatterplot displaying the correlation between predicted and experimental cell membrane permeability for the model described in Section 2.2.2. The points represent the mean of the predictive distribution from the prediction model used in PROTAC linker design based on the BRD4 and CRBN ligands ($R^2 = 0.683$, R = 0.831, and RMSE = 0.525). The upper and lower error bars correspond to the 70th and 30th percentiles of the predictive distribution, respectively.

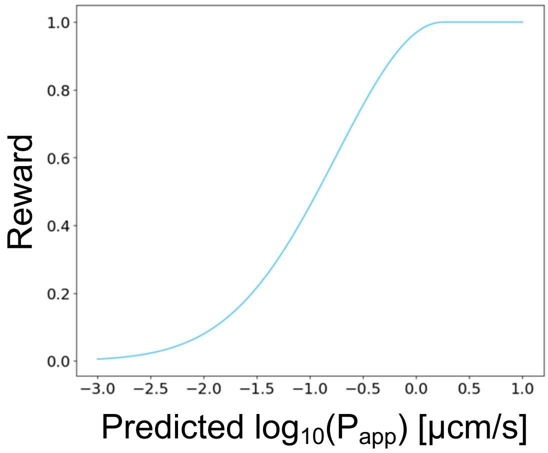

Figure S21. Left-sided Gaussian function with parameters $\mu = 0.25$ and $\sigma = 1$

Table S3. Filtering conditions

Target	Filter	Condition		
		Relaxed	Intermediate	Strict
Linker	Attachment point	✓	✓	✓
	Linker validation	✓	✓	✓
	Radical atom	✓	✓	✓
	Ring structure		✓	✓
	Branched structure		✓	✓
	Linker length			✓
	Linker similarity			✓
PROTAC	Alert substructure		✓	✓
	Specific substructure		✓	✓
	Prediction model AD			✓

Table S4. Alert substructures specified in the specific substructure filter

Alert Substructure
[C]#[C][!#6;!#1;!#15]
[!#6;!#1;!#15;!n;!o;!s][CH2][!#6;!#1;!#15;!n;!o;!s]
[S;!$(S(=O)=O)]~[N]
[!#6;!#1;!#15;!n;!o;!s;!$(S(=O)=O)][!#6;!#1;!#15;!n;!o;!s;!$(S(=O)=O)][!#6;!#1;!#15;!n;!o;!s;!$(S(=O)=O)]
[N]=[C]=[C]
[N]=[C]=[O]
[N]=[CH]
[C]=[S]
[n][!#6;!#1;!#15;!n;!o;!s]
